# Mesenchymal Stromal Cells for Enhancing Hematopoietic Engraftment and Treatment of Graft-Versus-Host Disease, Hemorrhages and Acute Respiratory Distress Syndrome

**DOI:** 10.3389/fimmu.2022.839844

**Published:** 2022-03-18

**Authors:** Olle Ringdén, Guido Moll, Britt Gustafsson, Behnam Sadeghi

**Affiliations:** ^1^ Translational Cell Therapy Research Group, Department of Clinical Sciences, Intervention and Technology (CLNTEC), Division of Pediatrics, Karolinska Institutet, Stockholm, Sweden; ^2^ Berlin Institute of Health (BIH) Center for Regenerative Therapies (BCRT) and Berlin-Brandenburg School for Regenerative Therapies (BSRT), Berlin, Germany; ^3^ Department of Nephrology and Internal Intensive Care Medicine, All Charité Universitätsmedizin Berlin, Corporate Member of Freie Universität Berlin and Humboldt-Universität zu Berlin, Berlin, Germany; ^4^ Department of Women’s and Children’s Health, Karolinska Institutet, Stockholm, Sweden

**Keywords:** cellular therapy, mesenchymal stromal cells (MSCs), immunomodulation, regeneration, hematopoietic engraftment, graft-versus host disease (GvHD), acute respiratory distress syndrome (ARDS), coronavirus-induced disease 2019 (COVID-19)

## Abstract

Mesenchymal stromal cells (MSCs) possess profound immunomodulatory and regenerative properties that are of clinical use in numerous clinical indications with unmet medical need. Common sources of MSCs include among others, bone marrow (BM), fat, umbilical cord, and placenta-derived decidua stromal cells (DSCs). We here summarize our more than 20-years of scientific experience in the clinical use of MSCs and DSCs in different clinical settings. BM-MSCs were first explored to enhance the engraftment of autografts in hematopoietic cell transplantation (HCT) and osteogenesis imperfecta around 30 years ago. In 2004, our group reported the first anti-inflammatory use of BM-MSCs in a child with grade IV acute graft-versus-host disease (GvHD). Subsequent studies have shown that MSCs appear to be more effective in acute than chronic GvHD. Today BM-MSC-therapy is registered for acute GvHD in Japan and for GvHD in children in Canada and New Zeeland. MSCs first home to the lung following intravenous injection and exert strong local and systemic immunomodulatory effects on the host immune system. Thus, they were studied for ameliorating the cytokine storm in acute respiratory distress syndrome (ARDS). Both, MSCs and DSCs were used to treat SARS-CoV-2 coronavirus-induced disease 2019 (COVID-19)-induced ARDS. In addition, they were also used for other novel indications, such as pneumomediastinum, colon perforation, and radiculomyelopathy. MSC and DSCs trigger coagulation and were thus explored to stop hemorrhages. DSCs appear to be more effective for acute GvHD, ARDS, and hemorrhages, but randomized studies are needed to prove superiority. Stromal cell infusion is safe, well tolerated, and only gives rise to a slight fever in a limited number of patients, but no major side effects have been reported in multiple safety studies and metaanalysis. In this review we summarize current evidence from *in vitro* studies, animal models, and importantly our clinical experience, to support stromal cell therapy in multiple clinical indications. This encloses MSC’s effects on the immune system, coagulation, and their safety and efficacy, which are discussed in relation to prominent clinical trials within the field.

## Introduction

Mesenchymal stromal cells (MSCs) were first described by Friedenstein and co-workers (1). MSCs are rare precursor cells that can be found in the bone marrow (BM) and all vascularized tissues in the body, such as adipose tissue (AT) and perinatal tissues (PT) (2, 3). They cannot regenerate and maintain a whole tissue compartment and are therefore not considered true stem cells (4). Many early studies used MSCs in regenerative medicine because of their capacity to differentiate into several mesenchymal tissue lineages, such as cartilage, bone, tendon, muscle and fat (5, 6).

However, it has recently become clear that MSCs exert their beneficial effects mainly through paracrine mechanisms (7). This entails both, cell contact-dependent and independent mechanisms, e.g. the secretion of various immunomodulatory and regenerative mediators that polarize the host immune system and promote tissue repair (8). MSCs suppress lymphocyte proliferation in mixed lymphocyte cultures (MLCs) *in vitro* and prolong skin allograft survival *in vivo* (9). Koc et al. demonstrated that autologous MSCs are safe to infuse in doses of 1-5x10^6^ cells/kg in patients who have undergone autologous hematopoietic cell transplantation (HCT) for breast cancer (10).

These investigations inspired us to apply MSCs in a 9-year-old boy with acute lymphoblastic leukemia in third remission, who had received HCT from an HLA-matched unrelated female donor. The boy had developed severe grade IV acute graft-versus-host disease (GvHD) that did not respond to cyclosporine, prednisolone, methylprednisolone, extracorporeal psoralen with ultraviolet light, Infliximab/Daclizumab. He was deteriorating, presented with bloody diarrhea 20 times/day, and had a bilirubin of 250 mmol/L. After 4 weeks we had expanded enough BM-MSCs from his mother to employ a dose of 2x10^6^ cells/kg. After MSCs infusion the patient had a dramatic response with normal stools and normal bilirubin within a week (11). This was followed by a BM-MSC pilot study in 8 patients with severe acute GvHD at our center (12) and a European multicenter study (13).

**Figure 1:**
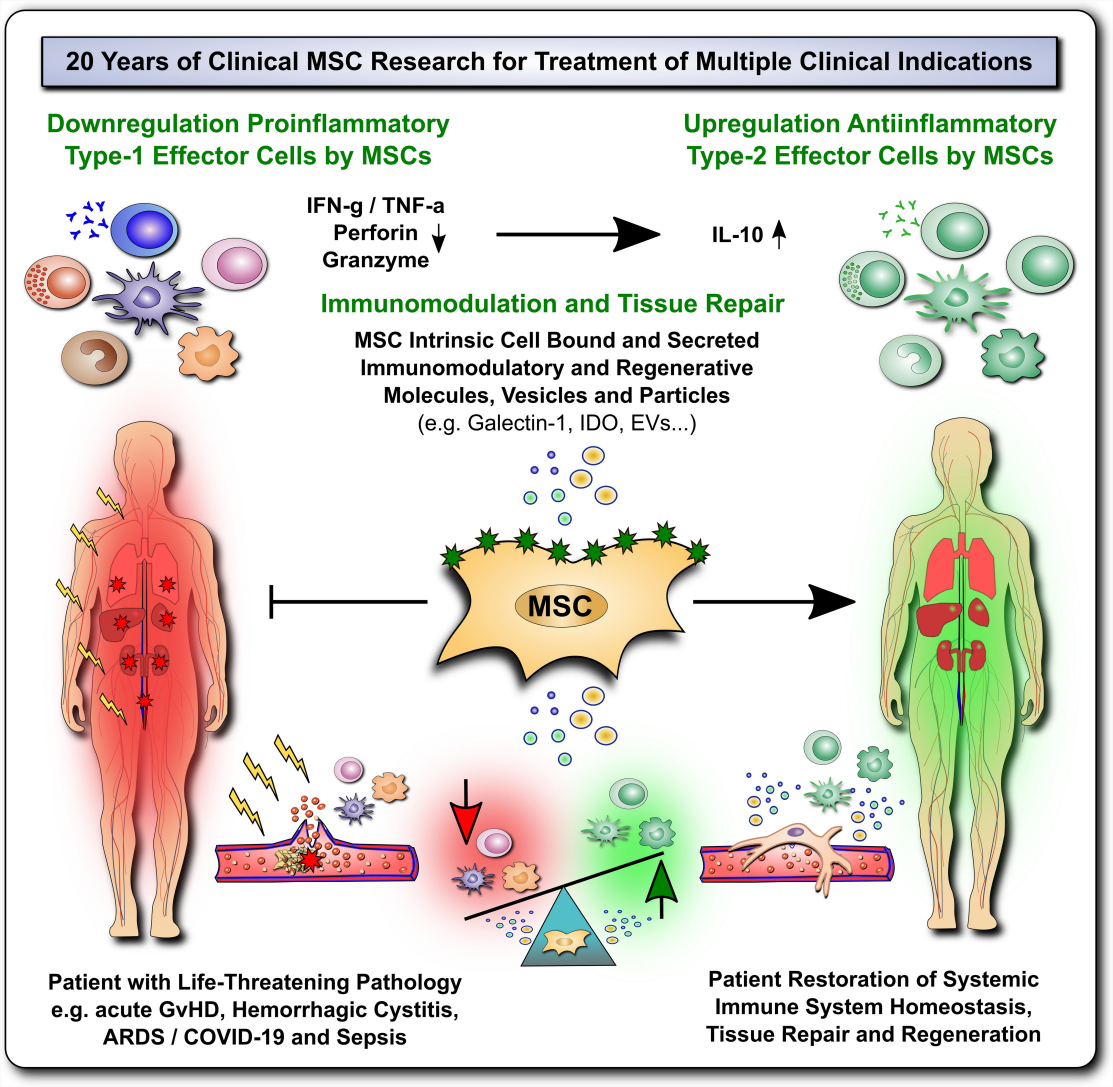
20 Years of Clinical MSC Research for Treatment for Multiple Life-Threatening Clinical Indications. The past 20 years of clinical mesenchymal stromal cell (MSC) research have shown that these cells can elicit profound immunomodulation and tissue repair upon therapeutic transfer into severely ill patients with multiple life-threatening clinical indications, such as acute Graft-versus-Host Disease (GvHD), Hemorrhagic Cystitis, Acute Respiratory Distress Syndrome (ARDS), Coronavirus 2019 Induced Disease (COVID-19) and Sepsis. This presumably occurs due to down-regulation of proinflammatory type-1 effector cells and upregulation of anti-inflammatory type-2 effector cells by MSCs through multiple MSC intrinsic cell bound and secreted immunomodulatory and regenerative mediators that restore and promote the patients' immune system homeostasis, tissue repair and regeneration.

MSCs have been investigated in a large number of trials for acute GvHD since then (14). For severe acute GvHD, MSCs were more encouraging in children than adults (13, 15). BM-MSCs are now registered as a therapy for acute GvHD in Japan and for children in Canada and New Zeeland (16). Despite preliminary encouraging findings, controlled trials did not show significant efficacy of using BM-MSCs for the treatment of steroid-refractory acute GvHD (17). In this review article, we summarize our scientific and clinical experience on the use of BM-MSCs and placenta-derived DSCs in different clinical indications in relation to prominent clinical trials (**Figure 1**), entailing studies in HCT, GvHD, hemorrhagic cystitis, acute respiratory distress syndrome (ARDS) and coronavirus-induced disease 2019 (COVID-19).

## MSC Immunomodulation and Immunological Properties

**Figure 2:**
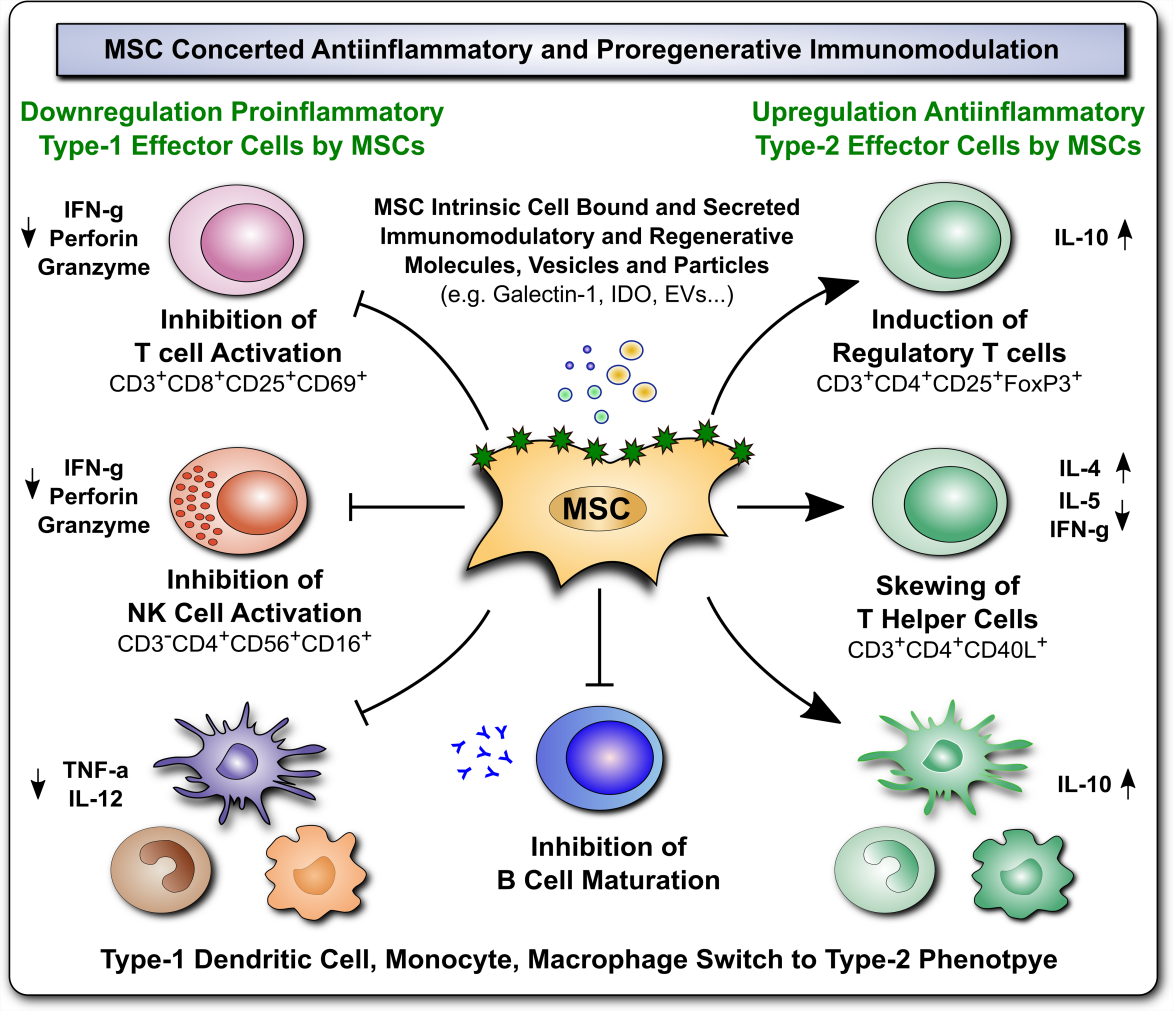
Immunomodulatory and Regenerative Properties of MSCs. Mesenchymal stromal cells (MSCs) employ a broad array of antiinflammatory and proregenerative immunomodulatory mechanisms, which are exerted either by MSCs directly, through intrinsic cell associated/bound, or secreted immunomodulatory and regenerative molecules, vesicles/particles (e.g. Galectin-1, indoleamine-2,3-dioxygenase (IDO), and extracellular vesicles, (EVs), respectively), or alternatively indirectly through MSCmediated favorable polarization of innate and adaptive immune responses (e.g. downregulation of proinflammatory type-1 effector cells, such as inhibition of T and NK cell activation, and upregulation of antiinflammatory type 2 effector cells, such as induction of regulatory T cells, skewing of T helper cells, and switch in type 1 dendritic cell, monocyte, and macrophage phages to type 2 phenotype, and inhibition of B cell maturation), that both lead to favorable changes in the predominant cytokine milieu (e.g. downregulation of proinflammatory mediators TNF-a, IFN-g, perforin and granzyme, but induction of antiinflammatory IL-10, in conjunction with secretion of multiple trophic and regenerative factors, not shown here) that altogether mediate beneficial tissue repair and proregenerative responses.

**Figure 3:**
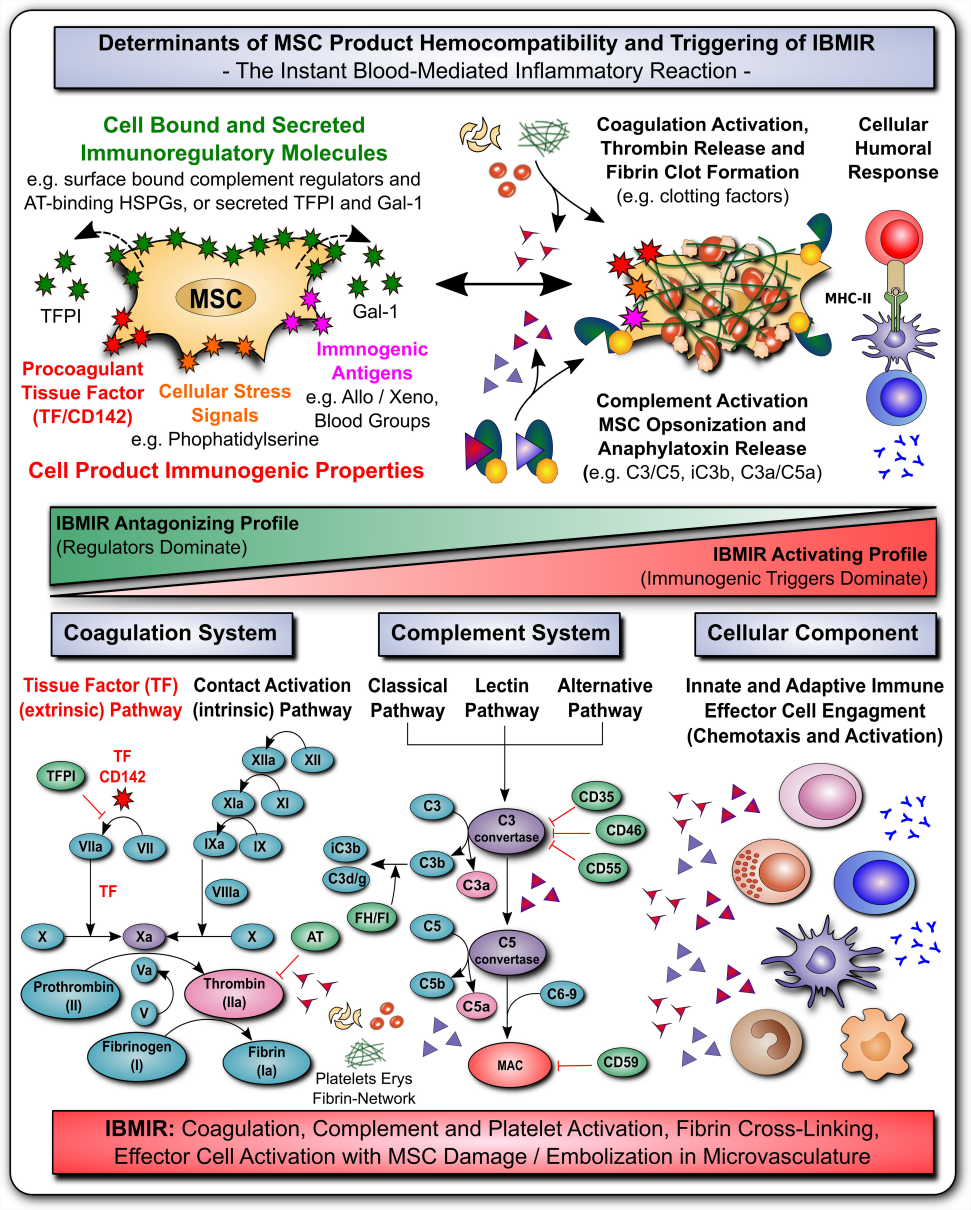
MSC Determinants of Immunogenicity, Hemocompatibility and Interaction with the Innate Immune Cascade Systems. **(A)** The safety and efficacy of infused mesenchymal stromal cell (MSC) products depends on their hemocompatibility profile and concomitant triggering of the instant blood-mediated inflammatory reaction (IBMIR). In analogy to their immunomodulatory features, the cells display a broad array of either regulatory elements or immunogenic triggering factors (e.g. cell bound or secreted regulators of complement and coagulation cascade and cellular immunity). A tight balance between triggering and regulatory elements is decisive for the triggering of IBMIR (incompatibility with blood) or prevention thereof (hemocompatibility). While multiple blood regulatory elements employed by MSCs prevent blood activation akin to mechanism employed by hemocompatible endothelial cells, therapeutic MSC products can also display varying levels of immunogenic triggers, such as highly procoagulant tissue factor (TF/CD142), cellular stress signals (e.g. cell surface exposure of complement and coagulation activating phosphatidylserine resulting from membrane asymmetry upon freeze-thawing), and immunogenic antigens (e.g. allo-, xeno-, and blood group antigens). If these triggers prevail over the regulatory elements, blood-incompatible MSC products that are introduced into the blood stream can trigger the IBMIR, entailing the activation of complement, coagulation, and cellular immune responses, which may compromise MSC product safety and functionality. **(B)** Considering the coagulation cascade, regulatory elements entail amongst others the secreted tissue factor pathway inhibitor (TFPI) and surface localized heparan-sulfate proteoglycans (HSPGs) that bind antithrombin (AT), which are both strong negative-regulators of the highly procoagulant thrombin (activator of fibrin and platelets). Thrombin is formed upon triggering of the clotting cascade by activation of the extrinsic tissue factor pathway of coagulation (initiated by conversion of factor FVII to FVIIa), or the intrinsic contact activation pathway of coagulation (initiated through conversion of FXII to FXIIa, e.g. upon blood exposure of highly negatively charged basement membrane contained collagen residues). Both arms of the coagulation cascade converge where FX is turned into FXa, that promotes the conversion of prothrombin to thrombin, which in turn elicits conversion of fibrinogen to fibrin, that will form the fibrin clot through cross-linking fibrin fibers, and incorporating activated platelets, erythrocytes, and nucleated white blood cells. **(C)** Considering the complement cascade, regulatory elements entail the cell surface bound complement regulators CD35, CD46, CD55, CD59 and the secreted complement regulators factor H and I (FH/FI), that can regulate the cascade at different steps, as indicated in the figure, and may thus prevent formation of the final membrane attack complex (MAC) albeit initiation of the earlier steps of complement cascade activation. Activation of the complement cascade can occur *via* the classical, lectin, and alternative pathways of coagulation, triggered among others *via* recognition of aberrant cell surface features (e.g. phosphatidylserine exposure upon freeze-thawing) or bound immunoglobulins *via* C1q (classical pathway), which is then transmitted through activation of the central complement components 3 and 5 (C3/C5), with concomitant formation of cell surface bound opsonins C3b/iC3b/C3dg and soluble chemotactic anaphylatoxins C3a and C5a, that can attract and activate various types of nucleated effector cells, such as T, NK, B cells and various phagocytes (e.g. PMNs, monocytes, macrophages and dendritic cells). **(D)** Considering the cellular component, as shown in **Figures 1** and **2**, MSC possess very potent immunoregulatory features to modulate adaptive and humoral branches of cellular immunity, that may however be compromised or skewed in an unfavorable direction if IBMIR-mediated killing of infused cells occurs to rapidly for the cells to exert their beneficial effects (e.g. induction of cellular humoral response and alloimunization in response to third-party cells). Overall, the triggering of IBMIR by infused therapeutic cell products may lead to coagulation, complement, and platelet activation, fibrin-cross-linking, and clot formation, with concomitant effector cell activation, and consecutive MSC damage and embolization in the microvasculature.

The immunomodulatory and immunological properties of MSCs are diverse and affect almost every major arm of the human immune system (**Figures 1**–**3**). They have been reviewed earlier (8). We here only briefly summarize this important aspect of MSC biology.

First of all, MSCs exhibit profound immunomodulation of both the adaptive and innate immune system, which is an integral part of their regenerative and therapeutic properties (8). Initial *in vitro* studies documented that MSCs’ inhibitory effect in alloantigen and mitogen-activated mixed lymphocyte culture (MLCs) did not depend on human leukocyte antigen (HLA)-compatibility between responder cells and MSCs (18). Interestingly, the cells also inhibit MLCs after differentiation to bone or cartilage (19). Indeed, it is a common property of different types of stromal cells from various tissue sources, including placenta-derived decidua stromal cells (DSCs) and fibroblasts, that these cells can inhibit MLCs (20–22). However, differences in intensity/quality of suppression may be apparent.

On a cellular level, CD4+ CD25+ regulatory T cells (Tregs) and IL-10 production are increased by both MSCs and DSCs (23–27). Type I dendritic cells decrease their TNF-α and IL-12 production when co-cultured with MSCs, and MSCs promote IL-10 secretion by LPS-stimulated type 2 dendritic cells. Both, MSCs and DSCs produce numerous immunomodulatory and immunosuppressive factors (8, 27), including HLA-G5 (28), prostaglandin E2 (23), several Galectin’s (e.g. Galectin-1) (29), and indoleamine-2,3-dioxygenase (IDO) (30). IDO inhibits T cells by conversion of tryptophan to kynurenine (31). MSCs can inhibit MLCs by soluble factors and also by direct contact with lymphocytes (27).

DSCs mainly suppress lymphocyte proliferation through both paracrine mechanisms and cell-to-cell contact (27, 32). Importantly, DSCs from the placental fetal membrane induce substantially stronger inhibition of MLCs compared to BM-MSCs and other sources of stromal cells from the placenta (33), which may also relate to their strong potency to modulate immune responses *in vivo* (7, 34). Apart from the secretion of soluble mediators, their direct contact-dependent effects include activation of the PD-1 pathway (35) and the activation of VCAM-1 and ICAM-1 (36). MSCs upregulate CD39 and adenosine production to suppress activated T cells (37) and they also induce Fas-mediated T cell apoptosis (38).

Sophisticated mechanistic studies by Galleu et al. have shown that allogeneic MSCs undergo perforin-dependent apoptosis by recipient cytotoxic T-cells after infusion, to reverse GvHD in a mouse model and this may also be part of their MoA in respective patients (39). Recipient phagocytes engulf apoptotic MSCs and produce IDO, necessary for suppression and to prevent GvHD (40). BM-MSCs produce exosomes with immunosuppressive and homing abilities (41). Exosomes from MSCs were also shown to be able to suppress and reverse acute GvHD (42). As opposed to BM-MSC, DSCs do not need IFN-γ licensing to be activated and show its immunomodulatory effect (43).

From an immunophenotypic point, MSCs are negative for common hematopoietic, myeloid, and endothelial markers (e.g. CD34, CD45, CD14, and CD31) and they are positive for a panel of commonly expressed markers (e.g. CD29, CD73, CD90, CD105, and CD166) (44, 45). Indeed, there is no specific commonly accepted surface marker for MSCs thoroughly established yet. DSCs have a similar immunophenotype as BM-MSCs (e.g. positive for CD29, CD73, CD105, and absence of CD34, etc) with only minor differences in surface antigen expression. These entail higher expression of PDL-1, PDL-2, and complement regulatory molecule CD55 (DAF, decay-accelerating factor) and procoagulant tissue factor (TF/CD142) (44). The latter two have an impact on hemocompatibility and triggering of the instant blood mediated inflammatory reaction (IBMIR; see below) (44, 46–49).

Initial studies suggested that MSCs are immune-privileged and do not induce strong immune stimulation (9, 19, 20). A recent review suggested that MSCs are rather immune evasive than truly immune privileged (50). MSCs express regular levels of HLA class I molecules and only low to absent levels of HLA class II. Both can be upregulated by inflammatory mediators, such as IFN-γ. Fas-ligand and co-stimulatory molecules, B-71, B-72, CD40, and CD40L are not expressed (20). DSCs only express HLA class I, and exposure to IFN-γ does not induce HLA class II expression (33).

Nonetheless, xenogeneic, and allogeneic MSCs/DSCs are rejected (51–53). After systemic infusion, neither MSCs nor DSCs are long-lived and are found in the circulation only shortly after infusion, partly due to a lung-entrapment effect (10, 53, 54). Only low levels of gene-marked MSCs were found in the BM of children with osteogenesis imperfecta (OI) (55). In a baboon model alloantibodies were induced by allogeneic MSCs (56). However, we did not detect any anti-HLA antibodies in 12 GvHD patients treated with BM-MSCs (57). Similarly, DSCs did not induce alloantibodies in patients with GvHD (58).

## MSC Hemocompatibility and Innate Immune Cascade Systems

The majority of clinical applications involving both MSCs and DSCs employ systemic infusion as a mode of clinical delivery (46, 59, 60). Thus, it is of crucial importance to study hemocompatibility aspects of the infused cell products (46–48, 61–66) and to understand their interaction with the innate immune defense cascade system, which are crucial for the processes of hemostasis and thrombosis. Therapeutic stromal cells can impact on existing pathology, such as hemorrhages, by modulating these cascade systems *in vivo* (60, 67–69). Thus, hemocompatibility aspects impact both safety and efficacy (**Figure 3**) (46–48, 61, 70).

The triggering of instant immune responses to stromal and other cell products infused into the bloodstream has been summarized under the term “IBMIR” – The instant blood-mediated inflammatory reaction (49). The intensity of IBMIR-triggering differs between stromal cell products from different sources and is influenced by several manufacturing parameters, such as their degree of *in vitro* expansion and cryopreservation. There are high variations between tissue sources and minimal expanded MSCs trigger less IBMIR than highly expanded cell products. Freeze-thawed cells trigger more IBMIR than fresh, culture-derived cells, which may correlate to some degree with their safety and efficacy profile (7, 45–47, 49, 62, 71–73).

The molecular mechanisms underlying the regulation of the innate immune cascade systems and their crosstalk with infused cells is highly complex and interlinked, involving a multitude of factors and crosstalk/feedback mechanisms that are hard to predict (70). Thus, hemocompatibility assessment of MSC products requires whole blood exposure *in vitro* and *in vivo* testing to understand the ultimate outcome (46–48). The complement, coagulation, and fibrinolytic cascades involve >30 soluble and cell bound molecules (**Figure 3**). This includes the primary cascade factors and a multitude of regulatory elements at different steps of the cascade, from the initial triggering or starting point to the final effector pathways, with the highly complex interaction between the three primary systems (70).

The coagulation system is primarily initiated/triggered *via* two effector pathways: 1) The Tissue Factor (TF/CD142) Pathway and 2) The Contact Activation Pathway, involving the recognition of blood exposed TF/CD142 and highly negatively charged collagen residues by coagulation factors VII and XII, respectively. We have studied in detail the pro- and anti-coagulant mechanisms/features employed by MSCs from different tissue sources (46). This includes their differential expression of highly procoagulant TF/CD142 (e.g. lowest to highest expression for BM-MSCs, PT-MSCs, and AT-MSCs, respectively) (46, 48) and collagens as typical pro-coagulant effectors, their expression of regulatory elements, such as tissue factor pathway inhibitor (TFPI) and prostacyclin (PGI2; a potent inhibitor of platelet activation), and surface expression of antithrombin-binding heparan sulfate proteoglycans (44, 49, 62, 74). Although principle generalizations can be made, each cell product may differ due to different underlying manufacturing processes, and may thus have to be assessed independently to study its distinct hemocompatibility profile (48). This is further impacted by the treatment indication and underlying coagulopathies/anticoagulation regimens (47, 62, 75–77).

Another key cascade system is the complement system, which is primarily triggered through three pathways: 1) The Classical Pathway (e.g. triggered by C1q), 2) The Lectin Pathway (e.g. triggered by MBL), and 3) The Alternative Pathway (“Tickover”). The complement system can recognize aberrant cell surface features of infused MSCs that are unfamiliar to the whole blood environment (45, 71, 72, 74, 78–81) when their complement regulatory features are insufficient to counterbalance activation (78, 80, 81). Thawed cells directly derived from cryostorage before clinical use can exhibit cell membrane asymmetry and phosphatidylserine exposure, which can be recognized by C1q/MBL (45, 71, 74). Cell surface binding of immunoglobulins of various specificities (e.g. towards immunogenic blood type ABO antigens, foreign donor alloantigen, or xenogeneic supplements/fetal calf serum) can promote complement activation (71, 74, 79). In addition, MSCs exhibit potent fibrinolytic properties through their expression of a diverse array of matrix metalloproteinases (46).

## Safety and Studies on Potential Side Effects of Stromal Cells

Stromal cells are used clinically in doses ranging from 1 to 10 x 10^6^ cells/kg. Three large meta-analyses including thousands of BM-MSC-treated patients found only a slight increase in body temperature upon MSCs infusion in a few patients (82–84). In several well-controlled clinical studies, there was no other major infusion-related toxicity. While administration of third-party MSCs can occasionally induce minimal allo-immunization in an immunocompetent host, no major severe adverse events and no major infusion-related toxicity have been reported with BM-MSCs (82–84).

Among patients receiving 88 infusions of placenta-derived DSCs, there were three transient, spontaneously reversible events (fever, dyspnea, and vertigo) (60). However, we did not find any thromboembolic events in patients treated with DSCs (60). In addition, in a comprehensive prospective safety and toxicity study in rats and mice using human DSCs, no thrombosis, organ damage, or dysfunction and toxicity on vital organs (*e.g.* liver, kidney, and lung) was observed with doses as high as 40x10^6^ cells/kg (53).

This may be due to highly optimized DSC application procedures, involving several important safety measures, such as removal of larger cell aggregates by filtration through a synthetic micromesh (70μm) before cell injection, heparinized syringes, prophylactic anticoagulation in patients and animals, and substitution of immunogenic AB-plasma with nonimmunogenic human serum albumin (7, 34, 46, 48, 60, 69, 71, 79).

Nonetheless, cases of thromboembolic events (e.g. compromised hemocompatibility, micro-and macro-thrombosis, and thromboembolism) in response to highly procoagulant TF/CD142 expressing MSC products in conjunction with suboptimal application routines have been reported in the past and call for increased caution when employing AT- and UC-derived MSCs products with high TF/CD142 expression (48, 85–89).

Furthermore, three fairly recent meta-analyses found no association with MSCs therapy and organ system complications, infections, deaths, or malignancies (82–84). Autopsy of 18 patients treated with BM-MSCs following HCT showed no ectopic tissue formation (45, 90) when treated with fresh-from-culture-derived or cryopreserved freeze-thawed MSCs (45, 71).

Various sources of MSCs and DSCs have been used for medical treatment and they have some differences in features, e.g. DSCs are only half the size compared to BM-MSCs and do not differentiate well to the bone, cartilage, and fat as do BM-MSCs (44, 54). This may reduce microvascular entrapment and the risk for ectopic tissue formation. Pilot studies and meta-analyses support the safety of both MSCs and DSCs. In contrast to pharmacological immunosuppressive drugs, stromal cells have very little if any side effects or toxicity.

## Co-infusion of MSCs in HCT

In 46 HLA-identical sibling transplants, MSCs were infused at the time of transplant (91). Graft failure was not seen in any of the patients and grades III-IV acute GvHD was noted in 15% of patients. Ball et al. co-infused MSCs in children given HLA-haploidentical grafts (92) and none of the patients experienced graft failure compared to 10% among retrospective controls. We infused MSCs to enhance the engraftment of HCT in seven patients (93). Three of them were re-grafted for previous graft failure. Neutrophil counts >0.5x10^9^/L and platelet counts >30x10^9^/L were both achieved at a medium of 12 days. All patients achieved 100% donor chimerism.

Co-transplantation of MSCs was compared to placebo in a prospective randomized study in HCT patients (94). Engraftment of neutrophils and platelets was similar in the two groups. There was a decreased risk of acute GvHD and an increased probability of leukemic relapse in the group receiving MSCs. Two children were given haploidentical MSCs and HLA-identical sibling HCT (95). The two patients with previous graft failure had prompt engraftment, no severe GvHD, and were in good condition two years after HCT.

Baron et al. suggested that transplantation of MSCs derived from umbilical cord blood (UCB) may prevent lethal GvHD without increasing the risk of leukemic relapse following HCT (96). In a pediatric study, parental haploidentical MSCs were used to promote engraftment of UCB grafts with a survival advantage relative to controls (97). In 13 children receiving UCB transplants, co-infusion of third-party MSCs was performed (98). Compared to historical controls there were no differences in hematological recovery or rejection rate. However, the rate of grade III-IV acute GvHD was significantly decreased in the MSC group compared to the controls (p=0.05).

Six children with aplastic anemia were given MSCs together with HLA-haploidentical or unrelated HLA-matched grafts (99). Acute GvHD grade II developed only in one case, but no chronic GvHD was documented. All were alive at a median follow-up of 15 months. In a pediatric study of UCB transplants, third-party UCB-MSCs were co-transplanted (100). Neutrophil engraftment was achieved with a median of 19 days in MSC-treated patients, as compared to 24 days in the historical controls (p=0.03). Acute and chronic GvHD were comparable between the two groups. Forty-four children with severe aplastic anemia were treated with haploidentical HCT and co-transplantation of MSCs (101). The incidence was 29% for grade II-IV acute GvHD and 15% for chronic GvHD and overall survival was 77%.

In a multicenter study, 35 children with aplastic anemia underwent HLA-haploidentical HCT with co-transplantation of donor-derived BM-MSCs (102). All had 100% donor engraftment and incidence of grade II-IV acute GvHD and chronic GvHD was 26% and 23%, respectively. Overall survival at 22 months was 86%. A combination of haplo-HCT and allogeneic MSCs was used for the treatment of severe aplastic anemia in 103 pediatric patients (103). Cumulative incidence of grade II-IV acute GvHD was 26% and grade III-IV acute GvHD was 7%. The cumulative incidence of chronic GvHD was 26% and overall survival was 87% at a median follow-up of 40 months.

A recent meta-analysis, including children as well as adults, showed improved survival in patients treated with MSCs as prophylaxis in HCT patients (104). A low MSC content of the HCT graft, as measured as MSCs expansion at the second passage, was correlated to transplant-related mortality and acute GvHD III-IV (105). Overall, these data indicate that MSC prophylaxis in HCT is associated with decreased graft failure, development of less acute but not chronic GvHD, and maybe an overall survival benefit.

## Treatment of Acute GVHD

Early studies paved the way for BM-MSCs as a treatment for severe acute GvHD (11–13). In a multicenter study, patients with steroid-refractory acute GvHD who had a complete response had a two-year survival of 53% in contrast to 16% among partial- and non-responders (13). There was a trend for a better complete response of 64% in pediatric patients in contrast to 47% in adults in this and other studies, and we thus put a focus on children in the following sections. Several studies confirmed the benefit using MSCs for children with severe acute GvHD (106–108). In a study by Prasad et al. commercially available MSCs (Prochymal™) were given to 12 pediatric patients with steroid-refractory severe acute GvHD, with a complete response in 7 children (58%). Survival at 100 days was also 58%. Two children had a partial response, and no response was seen in 3 children.

In a study by Ball et al. using BM-MSCs in 37 pediatric patients treated for steroid-refractory acute GvHD, the response was 65% and 3-year survival was 57% (109). In a mixed study of children and adults in several Brazilian centers response rate was 50% and survival was 20% in severe acute GvHD (110). Platelet-lysate-expanded MSCs were used for the treatment of steroid-refractory acute GvHD in 8 pediatric patients and 22 adults (111). Complete and partial response was seen in 88% of children compared to 50% in adults (p=0.099). Survival was 88% and 29% in the two groups, respectively (p=0.003).

Bonig et al. used BM-MSCs generated from pooled mononuclear cells from multiple donors and the overall response was 82% and 180-day survival was 64%. In children, the response was 77% and two-year survival was 77% (112). A Turkish study including 33 children found that MSCs induced a complete response in 18 patients (54%), a partial response in 7 (21%), and no response in 8 children (24%) (113). Two-year survival was 63% in patients with complete or partial response (75%).

In a multicenter study of 260 children and adults, who were randomized in a double-blind controlled study using Prochymal™ for the treatment of severe acute GvHD (114). The primary end-point, complete response at day 28, was the same in the two study groups. In a later analysis among patients with acute GvHD III-IV, MSC-treated patients had an overall response rate of 65% compared to 23% in the placebo-treated patients (p=0.05) (115). Children had a better overall response compared to adults.

Kurtzberg et al. used Remestemcel-L (Commercial BM-MSC) in 241 pediatric patients with steroid-refractory acute GvHD (116). The children were given 2x10^6^ MSCs/kg of Remestemcel-L twice a week for 4 weeks. Overall response at day +28 was 65% and 100-day survival was 82% among responders and 39% among non-responders (p<0.001). Meta-Analysis in children and adults showed that overall survival was positively correlated to MSC-dose (p=0.02) (104). Children treated with Remestemcel-L were compared to matched patients from the Mouth Sinai Acute GvHD International Consortium (MAGIC) (117). Clinical response after 28 days was 18/25 (72%) in the Remestemcel-L group compared to 13/27 (48%) among the retrospective controls (p=0.08). When children with high levels of biomarkers Reg3a and ST2 for severe acute GvHD were compared, the MSC group had better survival at 180 days, 64% as opposed to 10% in children treated with best available therapy (P=0.01), which included extracorporeal photopheresis, etanercept, infliximab, ruxolitinib, anti-thymocyte globulin, MMF, alemtuzumab, basiliximab, and tocilizumab.

DSCs were used to treat six pediatric patients with severe acute GvHD, grade III-IV and all six responded (118). Unfortunately, one of the patients, a teenage boy with Sickle Cell Disease (SS) died of severe brain hemorrhages a few months post HCT, but at time of HCT, he had severe sequelae due to untreated SS. Another child died of leukemia relapse. Four children are still alive and well with a Lansky score of 100% and five-year survival was 67%.

Overall, these data indicate partial effectiveness of MSCs and DSCs particularly in the treatment of severe acute GvHD in children with room for improvement. Our experience regarding DSCs in the pediatric population is limited, and further studies are needed (118). The outcome with DSCs in adults with acute GvHD is most promising (34, 46, 119). In mainly adult patients, we reported that with an improved handling of DSCs (7, 46), cell viability was median 95% and all 21 patients treated for severe acute GvHD responded with a one-year survival of 76% (34). Long-term survival among those patients at four years was 54%, which was not statistically inferior to the survival among 453 patients without severe acute GvHD undergoing HCT during the same time-period (119).

## Treatment of Chronic GVHD

The use of MSCs is much more limited in chronic GvHD than in acute GvHD and most pilot studies using MSCs for chronic GvHD were performed in adult patients (12, 120, 121). Transient effects on chronic GvHD were reported in four children using platelet-lysate-expanded MSCs (107). Only one child had a complete response (the others flared). A Meta-Analysis on the use of MSCs in 76 pediatric and adult patients reported an overall response of 66% with complete responses in 23% of the patients and overall survival of 64% (104).

We used DSCs to treat 3 pediatric patients with severe grade 3 chronic GvHD according to National Institute of Health criteria (54). The three children presented with GvHD in the skin, mouth, eyes, gastrointestinal tract, liver, lungs, joints, and fascia. Two of the children had a response in the liver with normalization of elevated liver enzymes. In one child esophageal varices disappeared. However, there was no overall improvement and grading after therapy sustained very severe grade 3 (54).

A polish study reported treatment of 9 pediatric patients with steroid-refractory acute or chronic GvHD (122). The children were given BM-, AT- or UC-MSC from 1 up to 6 infusions with an overall partial or complete response rate of 56%. Three patients with overlap syndrome showed amelioration, but the condition was not permanent.

A Phase II multicenter randomized, double blind controlled study was performed using UC-derived MSCs as prophylaxis against chronic GvHD (123). The two-year cumulative incidence of chronic GvHD in the MSC group was 27.4% as compared with 49% in the non-MSC-treated controls (p=0.02). After MSCs infusions, increasing memory B-lymphocytes and regulatory T cells were observed.

A meta-analysis including children and adults with GvHD treated with MSCs suggested improved survival using MSCs (104). In conclusion, the data on MSC and DSC treatment for chronic GvHD are much more scarce than acute GvHD, but preliminary studies indicate partial effectiveness.

## Treatment of Hemorrhages

Hemorrhagic cystitis occurs in up to 70% of recipients of HCT (124) and is classified as early-onset (within 72 hours after transplantation) or late-onset. Early-onset hemorrhagic cystitis is mainly caused by direct toxic effects of irradiation, cyclophosphamide, busulfan, and other cytotoxic drugs used for conditioning. Late-onset hemorrhagic cystitis may be due to viral infections, e.g. polyomavirus (BK and JC virus) and adenovirus. Transfusions are given to maintain platelet and hemoglobin levels. Hyperhydration and diuretics are given in stages beyond mild disease. For advanced hemorrhagic cystitis, there is no established therapy, but several interventions have been tried, including urethral catheterization, nephrostomy, dialysis, ligation of hypogastric arteries, cystectomy, and hyperbaric oxygen.

Stromal cells may be an attractive alternative and we used BM-MSCs to treat children and adults with hemorrhagic cystitis following HCT (67, 125). Among 10 patients with moderate-to-severe pathology 8 had a complete response after infusion of MSCs (68). Two patients with life-threatening hemorrhagic cystitis and several medical problems died of multi-organ failure (67). Tong et al. used UCB- MSCs in 13 pediatric patients with severe BK-virus-induced hemorrhagic cystitis (126). The children were given a median of 2 (1-3) MSC infusions with 8 cured cases. The copy number of BK virus DNA was 4.43x10^8^ ml before MSCs treatment and 2.67x10^8^ ml after infusion (ns).

Placenta-derived DSCs have a stronger effect on coagulation and hemostasis than BM-MSCs due to higher expression of procoagulant TF/CD142 and thus stronger reduction of the clotting time (44, 46). We treated 11 patients (median age 33 years, range 8-50 years; 4 on pain-requiring opiates) with grades III-IV hemorrhagic cystitis with DSCs (median 1 dose, range 1-4 doses) (69). In 5 patients hematuria disappeared within 5 days after DSC infusion and patients who received DSCs within three days after the start of hematuria had a shorter duration of hematuria and pain than those who were given DSCs later. One-year survival among the 11 patients with severe hemorrhagic cystitis following HCT given DSCs was 82%. These data are promising and presently we plan a prospective placebo-controlled study.

## Treatment of ARDS

Acute respiratory distress syndrome (ARDS) is a life-threatening lung condition with high mortality (127–129). The syndrome consists of acute hypoxic respiratory failure with bilateral pulmonary infiltrates associated with sepsis, pneumonia, shock, or aspiration.

Acute lung injury (ALI) and ARDS are characterized by rapid alveolar injury, inflammation, cytokine storm, and neutrophil accumulation. This causes pulmonary edema, leading to severe hypoxemia and impaired carbon dioxide excretion. ALI/ARDS may occur during the pancytopenia phase after HCT. The rationale to use stromal cells for ALI/ARDS is that these cells have a profound anti-inflammatory effect and first home to the lung after intravenous infusion (46, 54, 130). The hope is that MSCs ameliorate the ARDS-induced cytokine storm in the lungs.

We treated an adolescent boy with BM-MSCs in 2004 due to ARDS (131). The pulmonary infiltrates cleared, but the patient had multiple severe medical problems and died of multi-organ failure. We treated a 13-year-old boy with granulocyte transfusions, due to neutropenia after HCT. Subsequently, he developed ARDS and was treated with 1x10^6^ BM-MSCs/kg. He required ventilator assistance and died of massive aspergillosis pneumonia as revealed by autopsy.

AT-derived MSCs were used in a prospective randomized placebo-controlled study in 12 patients with ARDS (132). There was no difference in outcome between the two groups. In a phase I study, 3 patients each received 1x10^6^, 5x10^6,^ or 10x10^6^ cryopreserved BM-MSCs/kg as a single infusion for ARDS (133). The dose of 10x10^6^ MSCs/kg was selected for a randomized phase II safety study (134). Survival was the same in the MSC and placebo groups. MSC viability was low post-cryo-recovery (7, 47, 71, 135).

We treated a young adult, with DSCs, who developed α–streptococci septicemia during pancytopenia after HCT (136). With 15 L/min oxygen using a face mask and an oxygen saturation of 92%, he was in imminent need of ventilator assistance. He received 1x10^6^ DSCs/kg. After DSCs infusion oxygen saturation instantly increased to 98%. Requirements for oxygen decreased and the oxygen supply was discontinued 5 days later. Chest radiography normalized. The patient was discharged a few days later, is alive and shows 100% Karnofysky`s score.

The COVID-19 pandemic has led to the death of 5 million people worldwide with more than 245 million infections (University JH. Corona-Virus Resource Center 2021, available from https://coronavirus.jhu.edu/). Death by COVID-19 is mainly due to ARDS (137) and the pronounced coagulopathy leading to cardiovascular demise with lung- and multi-organ failure (47). Thus, particular attention should be paid to thromboprophylaxis, MSC product quality and mode of delivery, when employing experimental MSCs therapies for treatment of COVID-19 ARDS (47). ARDS is caused by a cytokine storm in the lungs, inflammation, neutrophil accumulation and alveolar damage (128). The main rational to use MSCs for treating ARDS is their strong anti-inflammatory effect and that MSCs home to the lung after intravenous cell infusion, which is one of the organs most strongly compromised by this pathology (9, 47, 54, 130). It may be reasoned, that the stronger antiinflammatory and regenerative properties/effects of specific MSC products, the better their therapeutic effect may be on ARDS (comparative studies lacking so far). Thus, it could be reasoned that DSCs may be favored over BM-MSCs, due to their stronger immunosuppressive and regenerative properties in treatment of acute immune-related pathology (7, 34, 46). Indeed, several MSC studies are registered to treat COVID-19-induced ARDS (47, 138–140). UC-derived MSCs were used in a double-blind pilot study including 24 patients with mild COVID-19-ARDS. Survival was 91% in the MSCs group compared to 42% among the controls (p=0.05).

DSCs were given to 10 patients with ARDS induced by COVID-19 (77). Inflammatory cytokines (IL-6 and CRP) significantly decreased after DSCs infusion. The median oxygen saturation level increased from 80% to 95% (P=0.012). Pulmonary infiltrates disappeared in all patients and 7 patients survived and were discharged. No toxicity or infusion-related adverse effects were seen. In a study from Toronto (Mattsson, J., personal communication), DSCs were used to treat 10 patients with ARDS induced by Covid-19. All 10 survived and were discharged from the hospital.

Exosomes derived from BM-MSC were used to treat COVID-19 induced ARDS in 24 patients. The exosomes were safe, and recovery was seen in 17 (71%) of the patients (141). A case study of 3 severe Covid-19 patients treated with concentrated secretomes of hypoxia-conditioned MSCs reported safety and beneficial effects (142).

## Discussion

Although MSCs were first explored to enhance hematopoietic engraftment, they are not established for this indication yet (10). However, MSCs are now registered for the treatment of acute GvHD in Japan and in pediatric patients in Canada and New Zeeland (16), but failed to get FDA approval in the US. It is now almost two decades since we treated the 9-year-old boy with life-threatening acute GvHD with BM-MSCs (11). We were lucky to choose a child as the first patient since an adult with such advanced GvHD would not have survived the four weeks it took to isolate and expand enough MSCs for therapy.

MSCs are not established as a treatment for acute GvHD in adults, because a prospective randomized study by Osiris did not meet the primary endpoint, response at 28 days after HCT (115). In this study, children had a better outcome than adults. Also, in the first multi-center study using MSCs for acute GvHD, children tended to have a better outcome than adults (13).

An early multi-center study also demonstrated a favorable outcome in children as opposed to adults (15). This was not found in a more recent meta-analysis, where children and adults had the same response rate to MSCs (14). There is conflicting data and another meta-analysis suggested that children had a better outcome by MSCs-therapy for acute GvHD than adults (143). Regardless of age, it seems that BM-MSC is not the optimal source of stromal cells for acute GvHD. DSCs have a stronger immunomodulatory effect compared to BM-MSC *in vitro* in MLC and *in vivo* in patients treated for acute GVHD (33, 34).

MSCs were more successful in acute than chronic GvHD (107, 120, 121). Indeed, MSCs have a better effect on acute inflammatory disorders. In contrast, in chronic GvHD, there is a strong fibrotic component in addition to the underlying inflammatory pathology, that seems to be rather unaffected by MSC treatment. However, we identified a good response to DSCs in patients with chronic GvHD of the liver (54). This may be due to that stromal cell home to the lung after intravenous infusion and are then distributed to the liver and spleen, where they may exert their beneficial effects more efficiently than in peripheral fibrotic tissues (54).

Recently, Ruxolitinib was approved for steroid refractory acute GvHD in adults and pediatric patients above 12 years of age (144). Overall response at day 28 was 62% in the Ruxolitinib group, which was better than 39% in the control group (p<0.001). Ruxolitinib was associated with side effects such as neutropenia, thrombocytopenia, anemia, and infections. The study only included 5 children treated with Ruxolitinib. Ruxolitinib was not so encouraging in children with acute GvHD (145). This may be due to that children have different pharmacokinetics than adults and probably require higher dosages/kg.

Most studies employing MSCs reported that the treatment is safe (12, 115, 116, 146). Similarly, DSCs also seem safe (60). The safety of MSCs therapy is supported by three recent meta-analyses (82–84). A slight increase in temperature was noted following MSCs infusion, but no serious adverse events. This distinguishes stromal cells from many other immunosuppressive drugs, which typically report several adverse events (144). When MSCs are infused systemically, attention should be paid to differing hemostatic/procoagulant properties from different tissue sources, resulting from their differential expression of highly procoagulant TF/C142. A few cases of thromboembolism were reported (7, 46–48, 85–88). The risks for thrombosis can be minimized using well-characterized, high-quality, GMP-compliant MSC products (46, 48), with proper hemocompatibility screening prior to clinical use (7, 46–48). Considering the choice of MSC products for specific indications and mode of delivery, potential benefits need to be weighed against potential side effects (7, 46, 62).

MSCs’ procoagulant properties may be beneficial in the treatment of hemorrhages. MSCs and DSCs exhibit strong modulatory properties on coagulation and hemostasis and these effects are more profound for DSCs than BM-MSCs (44). Both cell types were used successfully to treat hemorrhages following HCT (67–69, 125, 126). BM-MSCs stopped gastrointestinal hemorrhages in a patient refractory to platelet transfusions. Despite encouraging studies, MSCs have only been used to treat hemorrhages in a few reports to date.

For hemorrhagic cystitis, both BM-MSCs as well as DSCs seem to be a superior option, compared to conventional treatments, as these traditional interventions are associated with high morbidity and mortality (147). As shown in our pilot studies, treatment with BM-MSCs and DSCs is an attractive alternative (67–69, 125). DSCs exhibit a stronger effect on coagulation and hemostasis (44, 46) and it seems to be an attractive option to use them in future studies, especially in the pediatric population when a child suffers from severe hemorrhagic cystitis stage III-IV. MSCs also have the potential to be used to stop fatal gastro-intestinal hemorrhages in very old patients with severe co-morbidities, where surgical intervention cannot be undertaken (125).

A concern with BM-MSCs has been possible ectopic tissue formation, because these cells differentiate well into cartilage, fat, and bone, among other mesenchymal tissues (6, 90, 115). Analysis of human autopsy material found no ectopic tissue following MSC infusion (90). DSCs may be even safer because they are half the size with a lower tendency for microvascular entrapment, and they do not differentiate well into cartilage and bone (44, 54).

Cell dosing and cryopreservation may be decisive factors in the safe and efficient clinical use of stromal cells (7, 45, 46, 71, 79). A wide range of MSCs cell doses were used safely, from 0.5 to 10 x 10^6^ cells/kg (46, 148, 149). Wilson and co-workers tested three different doses, 1x10^6^, 6x10^6^ and 10x10^6^ MSCs/kg and selected 10x10^6^ cells/kg in a prospective study for ARDS (133, 134). In the latter study, cell viability varied between 36% and 85% post thawing for clinical use, and low viability may have compromised clinical efficacy (135).

However, with optimal handling both fresh and freeze-thawed cells may have similar viability and immunomodulatory effects (Sadeghi B. et al. to be published) (71, 73). Indeed, the full impact of cryopreservation and freeze-thawing on MSCs’ therapeutic efficacy is controversially discussed in the moment, since several studies also reported that both fresh and freeze-thawed MSCs have similar immunomodulatory properties in preclinical models (150–154). Some recent studies even argued that frozen cells may be more advantageous since recognition of apoptotic cells by the host immune system may be part of their MoA (39).

With therapeutic DSCs, the median cell viability was more than 95% and cell batches with high viability had a better clinical response in treatment of severe acute GvHD (7, 34). Fresh BM-MSCs performed better than freeze-thawed cells (45). Thus, the highest possible cell viability should be aimed for, but above 80% may be sufficient. A cell dose of 1x10^6^ cell/kg seems to be effective in many clinical trials.

Many different sources of stromal cells were explored during the past decades (7, 46, 48). Most experience stems from BM-MSCs, but this requires rather invasive/painful harvesting by aspirating BM from patients or voluntary donors with limited material for cell expansion. In contrast, AT-derived MSCs are popular because they can be easily sourced from leftover material obtained during plastic surgery. In addition, perinatal and endometrial tissue sources (155), such as placenta, umbilical cord matrix, umbilical cord, or menstrual blood are non-invasive sources, with excellent expansion potential and high potency, including DSCs (33).

Our pilot studies indicate that DSCs are superior for inhibition of alloreactivity and have stronger paracrine activity compared to other sources of MSCs, which makes them valuable for immunosuppression. In contrast, a major advantage of BM-MSCs is that they differentiate very well into fat, cartilage, and bone (156), and therefore may be more valuable for tissue engineering and in regenerative medicine.

Due to the ongoing SARS-CoV2 coronavirus pandemic, where ARDS has been a frequent cause of death, many studies were registered for this indication using various sources of MSCs (47, 138). Two randomized placebo-controlled studies using AT- and BM-MSCs failed to prove efficacy for ARDS (132, 134). So far, PT-derived MSCs and DSCs seem to be promising for ARDS induced by COVID-19 (77, 140, 157, 158). Regarding DSCs a prospective randomized placebo- controlled trial is needed. Overall, more data and efficacy studies are needed, before ARDS can be established as an indication using MSCs (47).

MSCs are mainly used to treat acute GvHD, but not as extensively in chronic GvHD. Recent studies show that it may be possible to reverse severe acute GvHD by using exosomes from MSCs (42). Other indications where MSCs may be useful, such as pneumomediastinum, colon perforation, and radiculomyelopathy have been explored in adult HCT patients so far (67, 159). In children with osteogenesis imperfecta, HCT combined with MSCs treatment showed donor-osteoblast engraftment new dense bone, increased bone mineral content, and improved growth velocity (55, 160–162). In addition, patients who have undergone HCT for Hurler’s disease and metachromatic leukodystrophy were treated with MSCs to enhance enzyme production (163). Among children with metachromatic leukodystrophy, 4 of 5 had improvement in nerve conduction velocity.

## Conclusion

In this paper, we have discussed the use of MSCs to enhance hematopoietic engraftment, and as treatment for chronic and acute GvHD, hemorrhagic cystitis and ARDS. The later three pathologies are characterized by high levels of proinflammatory mediators. MSCs may be of particular use here, since they have potent immunomodulatory and regenerative effects, with a favorable safety profile compared to other conventional immunosuppressive drugs. The application of BM-MSCs and DSCs appears to be safe, although their procoagulant properties need to be anticipated for clinical use. Further improvements in clinical efficacy by stromal cells therapy may be achieved by optimizing the cell production process and by using stromal cell derived exosomes and extracellular vesicles instead of whole cell therapy. Product improvements may be achieved by optimizing cell viability and cryopreservation, the mode of cell delivery, innovative cell engineering concepts, and more potent products, such as DSCs. In the future, many different types of MSCs products tailored to specific clinical indications, but also exosomes derived thereof, may be used for several inflammatory disorders. Primary indications for MSCs and DSCs include among others acute GvHD and hemorrhages (e.g. hemorrhagic cystitis) and ARDS, but many other indications, e.g. osteogenesis imperfecta, rheumatic inflammatory disorders, cardiovascular diseases, sepsis and septic shock, inflammatory bowel disease, multiple sclerosis, neuroinflammation and other acute inflammatory disorders may join the line of established therapies (164, 165).

## Data Availability Statement

The original contributions presented in the study are included in the article/supplementary material. Further inquiries can be directed to the corresponding authors.

## Author Contributions

OR wrote the first draft of the article, complemented and revised by GM, BG, and BS. All authors contributed to the article and approved the submitted version.

## Funding

The study was supported with grants from the Swedish Cancer Foundation (CAN 2018, 419) and the Cancer Society in Stockholm (111293). OR was the recipient of a Distinguished Professor Award from the Karolinska Institutet. GM’s contributions were made possible by funding from the German Federal Ministry for Education and Research (BMBF) and German Research Foundation (DFG) through the Berlin Institute of Healthy (BIH)-Center for Regenerative Therapies (BCRT) and the Berlin-Brandenburg School for Regenerative Therapies (BSRT, GSC203), respectively, and in part by the European Union’s Horizon 2020 Research and Innovation Program under grant agreements No 733006 (PACE) and 779293 (HIPGEN). We acknowledge support from Frontiers Immunology and the DFG and the Open Access Publication Fund of Charité – Universitätsmedizin Berlin.

## Conflict of Interest

The authors declare that the research was conducted in the absence of any commercial or financial relationships that could be construed as a potential conflict of interest.

## Publisher’s Note

All claims expressed in this article are solely those of the authors and do not necessarily represent those of their affiliated organizations, or those of the publisher, the editors and the reviewers. Any product that may be evaluated in this article, or claim that may be made by its manufacturer, is not guaranteed or endorsed by the publisher.
